# Engineering large animal models of human disease

**DOI:** 10.1002/path.4648

**Published:** 2015-11-28

**Authors:** C Bruce A Whitelaw, Timothy P Sheets, Simon G Lillico, Bhanu P Telugu

**Affiliations:** ^1^ The Roslin Institute and Royal (Dick) School of Veterinary Science, Easter Bush CampusUniversity of EdinburghEdinburghEH25 9RGUK; ^2^ Animal Bioscience and Biotechnology Laboratory, ARSBeltsvilleMD20705USA; ^3^ Department of Animal and Avian SciencesBeltsvilleMD20742USA

**Keywords:** CRISPR, gene editing, livestock, pathology, pigs, SCNT, TALEN, ZFN, zygote

## Abstract

The recent development of gene editing tools and methodology for use in livestock enables the production of new animal disease models. These tools facilitate site‐specific mutation of the genome, allowing animals carrying known human disease mutations to be produced. In this review, we describe the various gene editing tools and how they can be used for a range of large animal models of diseases. This genomic technology is in its infancy but the expectation is that through the use of gene editing tools we will see a dramatic increase in animal model resources available for both the study of human disease and the translation of this knowledge into the clinic. Comparative pathology will be central to the productive use of these animal models and the successful translation of new therapeutic strategies. © 2015 The Authors. *The Journal of Pathology* published by John Wiley & Sons Ltd on behalf of Pathological Society of Great Britain and Ireland.

## Large animal models of disease

As the world population expands in number and increases in wealth, with people living longer than before, demands on the medical community to increase its arsenal of disease treatments are relentless. Although much debated, animal studies remain central to many regulatory systems as a safety checkpoint for testing new treatments – whether based on drugs, genetic solutions, or regenerative processes 1, 2, 3, 4. Additionally, prior to this late step in the development of a new treatment, research studies in animals often play a crucial role in providing both understanding of the disease and associated pathology, and identifying the target event in the disease to which the treatment is directed. By far the most utilized mammal in both of these phases is the laboratory mouse, and it is without question that studies in mice have dramatically accelerated our ability to treat disease. Nevertheless, as highlighted in numerous other reviews in this issue of *The Journal of Pathology*, mouse data can be inadequate in its ability to translate scientific progress from ‘bench to bedside’.

Significant differences between mouse and man, including physical size, limit the mouse as a model of human disease. An often cited example is that of cystic fibrosis, where mice carrying mutations of relevance to humans do not show the full panoply of symptoms associated with human cystic fibrosis 5, 6, 7, 8; the same is true for other diseases such as Lesch–Nylan syndrome 9 and Huntington's disease 10, 11. As a result, focus is increasingly directed to larger animals, with dogs and pigs seeing greatest use 12 in addition to primates 13. For many years, medical advances have been restricted by the availability of appropriate model species carrying disease conferring mutations. This small repertoire of naturally occurring diseases has been augmented by transgenic approaches, and good models have emerged, for example the GIPR (dn) 14 and INSC94Y 15 diabetic pigs. The considerable progress achieved since the first engineered large animal model of human disease was reported 16 has been reviewed 17, 18, 19. A recently developed set of tools, commonly termed gene editors (reviewed in refs 20–22), now allow those who want to understand disease processes or develop novel treatments to choose the most appropriate animal species for their studies.

## Gene editing with designer nuclease editors

Gene editors are site‐specific nucleases that introduce double‐strand breaks (DSBs) at specific loci within the genome. The engineered DSBs trigger DNA repair by two competing pathways, non‐homologous end joining (NHEJ) or homology‐directed repair (HDR), which facilitate the generation of knockout and knock‐in animals, respectively. Currently, there are four groups of gene editors available. The first are the meganucleases, which remain unpopular due to difficulties in production and limitations in target site selection 23. The other three groups – zinc finger nucleases, transcription activator‐like effector nucleases, and clustered regularly interspaced palindromic repeats (CRISPR) and CRISPR associated 9 (Cas9) nuclease – are seeing rapidly increasing use in animals.

### Zinc finger nuclease (ZFN)

ZFNs are adapted from the eukaryotic zinc finger class of transcription factors and utilize Cys_2_‐His_2_ DNA‐binding motifs for target recognition 24. The zinc finger (ZF) is one of the most common DNA binding motifs in mammals 25, with a single finger interacting specifically with a triplet of nucleotides. ZFNs are assembled by combining three to four zinc fingers in tandem, recognizing 9–12 base pairs of sequence, respectively, and tethering one half of the catalytic domain of the obligate dimeric endonuclease FokI 24. Thus, ZFNs are used in pairs that bind sequences on opposite DNA strands to facilitate dimerization of FokI to catalyse a DSB in the target DNA. This requirement for targeting two opposite strands in close proximity, as well as the use of heterodimeric FokI endonucleases, offers an advantage towards increasing site specificity and mitigating off‐target concerns 24, 26. The design of ZFNs is constrained by a requirement for high GC content, recognition of triplets, and an obligate requirement for a short spacer sequence. These requirements make rational design and assembly of ZFNs a somewhat daunting task for most laboratories 27. Additional bottlenecks include the unpredictability of ZFN efficiency, requiring intensive pre‐screening of several ZFNs and targeting sites 28. The oligomerized pool engineering strategy 29, 30 and context‐dependent assembly (CoDA) 31 have been developed to overcome these deficiencies. In large animals, including pigs, ZFNs have been used successfully for editing the genome.

### Transcription activator‐like effector nuclease (TALEN)

Similar to ZFNs, transcription activator‐like (TAL)‐effector modules are found naturally, being used by *Xanthomonas* bacteria to specifically bind host DNA and modify metabolism in favour of bacterial propagation 32. However, unlike zinc fingers that bind three nucleotides, each TAL module binds to a single nucleotide. TAL modules consist of 34 amino acids with residues at positions 12 and 13 (repeat variable diresidue; RVD) conferring DNA recognition. Based on the target sequence, it is possible to choose appropriate RVDs, assemble a modular array, and fuse to FokI to generate a TALEN 33, 34, 35, 36, 37, 38. As with ZFNs, TALENs are utilized as pairs with FokI dimerization in a spacer region. The relative ease of TALEN design and assembly has been illustrated by a recent publication in which a library of TALENs was assembled to target 18 700 human protein coding genes 39. For the same reasons, TALENS have been utilized to edit porcine, sheep, and cattle genomes 21, 40, 41, 42. Similar to ZFNs, off‐target cleavage remains a concern, which is mitigated by the use of obligate heterodimer FokI nucleases 43.

### Clustered regularly interspaced palindromic repeats (CRISPR) and CRISPR associated 9 (Cas9) nuclease

The CRISPR/Cas9 system evolved in archaea and eubacteria as an RNA‐based adaptive immune system to detect and cleave invading viruses and plasmids 44, 45. Currently, the most commonly used CRISPR/Cas9 system is a modified version of that used by *Streptococcus pyogenes* and consists of a guide RNA and Cas9 endonuclease. These two components form a complex, with a 20‐nt section of the guide sequence determining target identity via Watson–Crick base pairing, followed by cleavage by Cas9 to create a DSB. In a recent landmark publication, five genes were simultaneously targeted by the CRISPR/Cas9 system 46 in a mouse model.

The relative ease of design and manipulation, due to the requirement for a single as opposed to two recognition sites, makes the CRISPR/Cas9 system a widely used and desirable editor. However, off‐target cutting remains a major concern for this system 47. In this regard, modifications of Cas9 nuclease are critical for overcoming off‐target concerns. Single‐strand nickase activity 48, Cas9–FokI fusion nucleases 49, and split dimerizable Cas9 50 offer the potential to reduce the off‐target concerns of the CRISPR/Cas9 system. Taken together, the CRISPR/Cas9 system has proven to be an easy, versatile system, and currently the most widely used gene editor.

## Gene editors engineer genetic variation

**Figure 1:**
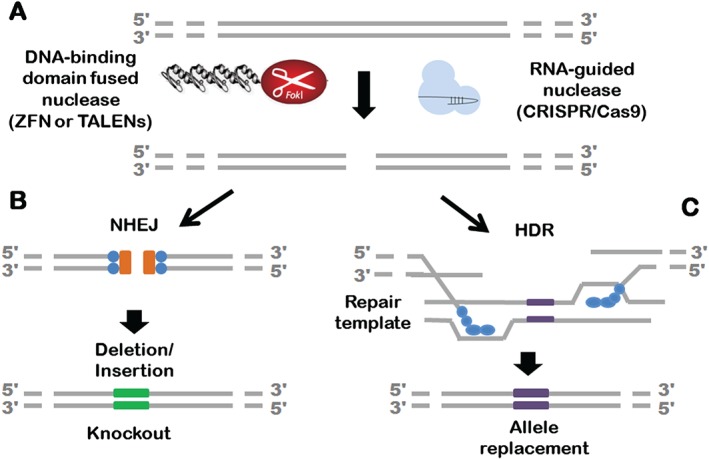
Site‐specific nuclease (SSN)‐mediated gene targeting. (A) Double‐stranded breaks (DSBs) at the target site can be induced by two classes of SSNs: either nucleases fused to a DNA‐binding domain, eg ZFN and TALENs (left), or an RNA‐guided nuclease (CRISPR/Cas9; right). (B) KU80 proteins bind the resected ends to initiate an error‐prone non‐homologous end joining (NHEJ) pathway, resulting in the potential introduction of insertions or deletions of a few nucleotides at the cut site (indels) and the generation of a premature stop codon effectively knocking out the allele. (C) Conversely, the DNA strands can undergo repair by homology‐directed repair (HDR). In this case, the DNA at the cut site undergoes end resection; binds to Rad51 proteins, initiating strand invasion of the repair template (either a single‐ or a double‐stranded DNA repair template); and allows high fidelity repair and precise editing, and replacement of alleles.

Following editor activity, DSB repair is carried out by one of two main pathways (see Figure 1). Non‐homologous end joining or NHEJ (a relatively broad term comprising of canonical‐ and alternative NHEJ pathways mediated by DNA ligase IV and I/III, respectively) is the major repair pathway that is active in all phases of the cell cycle. Canonical NHEJ (c‐NHEJ) is the predominant pathway following Cas9‐mediated DSB in the murine genome, with experimental efficiencies reaching 20–60% 40, 51, 52. In brief, following DSB (Figure 1A), Ku proteins bind the cut ends; the Ku:DNA complex serves as a platform for other components of the NHEJ repair pathway such as nuclease, polymerase, and DNA ligase IV to dock and initiate repair (Figure 1B). DNA ligase IV has limited sequence preference and can ligate DNA strands across gaps, ligate incompatible ends, and even ligate single strands. This mechanistic flexibility of DNA ligase IV in the NHEJ pathway results in a significant variability of processed ends resulting in insertions or deletions (indels) around cut sites. When such indels are introduced into the open reading frame of a functional gene, the result can be a frameshift and functional knockout of the gene. As NHEJ is both error‐prone and the predominant repair pathway following DSB, frequently no additional manipulation of the cell is required for ablation of target gene function besides introduction of editors.

When functional knockout is not the desired goal, more precise modifications of the genome including introduction of point mutations, modification of codons, introduction of reporters or replacement of alleles can be performed. Such precise modifications are dependent on HDR (Figure 1B). The frequency of HDR in mammalian systems is extremely low, but can be improved by several orders of magnitude with the introduction of a DSB at the target site 53. Following a DSB, repair by HDR is dependent on the occurrence of 5'‐end resection and the generation of single‐stranded 3' ends 54, 55. The single‐stranded 3' ends serve as a scaffold for assembly of Rad51 filaments instead of Ku proteins for initiating repair. The Rad51 element directs strand invasion of a homologous DNA template, while the 3' end serves as a primer for repair synthesis 56. It should be noted that even with Cas9‐induced DSBs, the efficiency of HDR is only 0.5–20% in mouse systems 40, 51, 57 because such repair takes place within the context of a competing NHEJ pathway. By contrast to NHEJ that takes place throughout the cell cycle 58, HDR is only functional in the S and G2 phases 48, 51, 52. Binding of Ku proteins to exposed ends prevents end resection and biases the pathway to NHEJ; suppression of Ku proteins has been shown to significantly improve the rate of HDR in gene editing experiments 30, 59. Likewise, inhibition of ligase IV by the synthetic inhibitor SCR7 results in up to a 19‐fold increase in the rate of Cas9‐mediated HDR in mammalian cell lines 59. Encouragingly, the use of SCR7 in the context of porcine cells and embryos has been in line with the findings from mice, and it is expected that SCR7 will find greater applicability in HDR experiments in porcine systems (Telugu et al, unpublished results).

Contrary to DSBs, single‐strand breaks and gaps are preferentially repaired by the HDR pathway. Using D10A Cas9 nickase, a targeted nick or gap will, in the next round of DNA synthesis, become a DSB and initiate repair by HDR in mammalian cells. Additional evidence for this in mammalian systems comes from the use of site‐specific nickases, such as meganucleases 60, zinc finger nucleases 61, 62, 63, and CRISPR/Cas9 45, 48. For the purposes of gene editing, the use of nickases that generate a single‐strand nick has the advantage that nicks are not repaired by c‐NHEJ. However, the induction of HDR from nicks is usually much less efficient than that from DSBs. In this context, HDR is still expected to be advanced by the use of DSB and inhibition of the c‐NHEJ pathway.

## Engineering large animals: SCNT versus zygote injections

**Figure 2:**
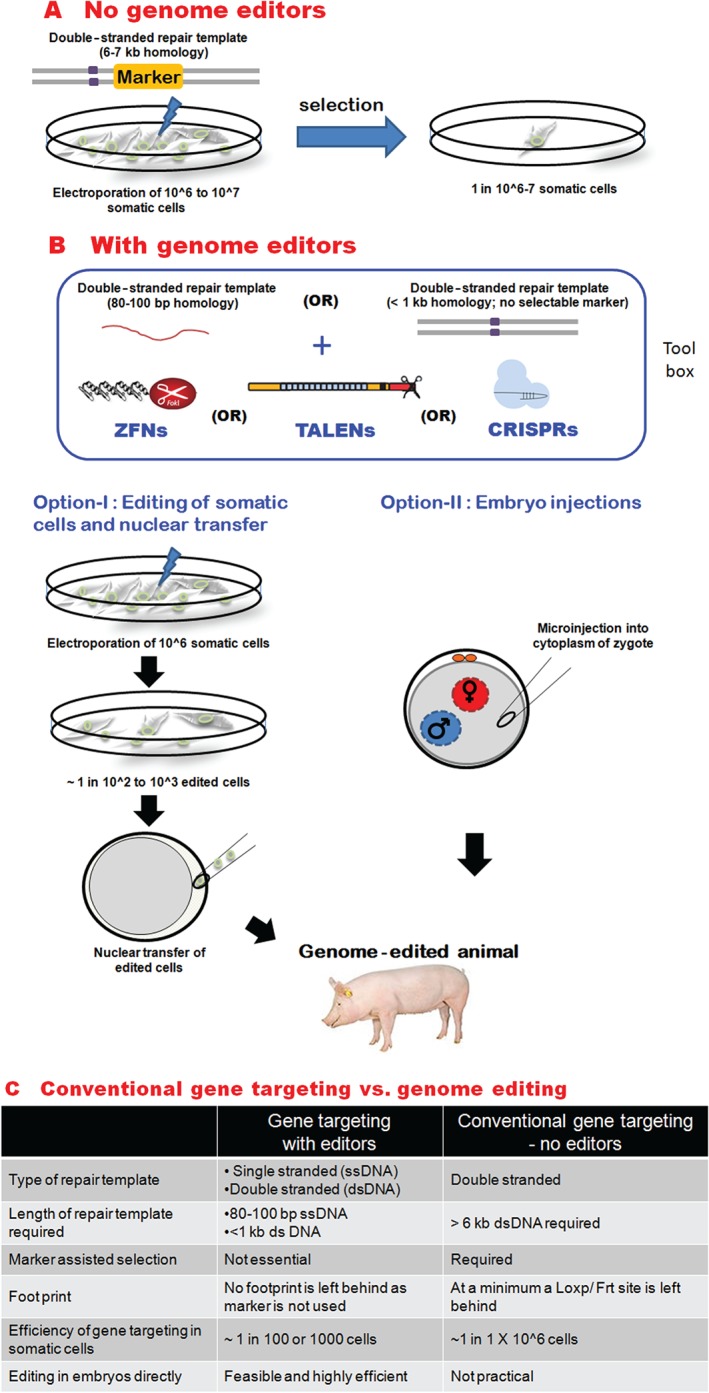
Genome editing in pigs. (A) For gene targeting without editors, a double‐stranded DNA targeting vector with the intended gene modification (purple square) with a selectable marker, eg a neomycin resistance cassette, is used and the cells that survive the selection are used for gene targeting. (B) For targeting with editors, a selection of tools such as zinc finger nucleases (ZFNs), TAL‐effector nucleases (TALENs), and clustered regularly interspaced palindromic repeats (CRISPR)–CRISPR associated 9 (Cas9) nuclease can be used to introduce double‐strand breaks in the genome. When used by themselves, the editors will generate knockout of genes. In combination with either a single‐stranded or a double‐stranded DNA as the repair template, the editors will facilitate gene targeting. The editors, with or without the targeting vectors, can be electroporated into somatic cells and used as donors for nuclear transfer or cloning to generate edited animals (Option‐I), or microinjected into the cytoplasm of embryos (Option‐II). (C) A comparison of conventional gene targeting and genome editing with editors is shown.

The majority of genetically engineered livestock are pigs and in this species, somatic cell nuclear transfer (SCNT) or cloning remains by far the most popular production method (see Figure 2). The technique involves the generation of somatic cells (typically porcine fetal fibroblasts) carrying the intended genetic modification and using these cells as donors in cloning experiments (Figure 2B). For cloning, the metaphase II plate within the oocyte comprising the genetic material is removed, and the genetically modified cell fused with the enucleated oocyte to restart embryo development. Reconstituted oocytes are typically transferred into the oviducts of recipient animals. Editor technology can be easily applied to create either NHEJ‐ or HDR‐driven mutations within the donor cell in vitro (Figures 2A and 2B). Usually a pre‐screening or selection strategy is used to enable enrichment for cells carrying the desired mutation.

The major advantage of SCNT over direct embryo injection with editor reagents is the predictable genotype of piglets and the ability to generate clonal lines of edited animals. However, SCNT suffers from serious disadvantages, such as the relatively low viability of reconstituted embryos and, consequently, pregnancy losses following embryo transfer. Therefore, a high number of reconstituted embryos, normally in the range of 100–150 embryos, are typically transferred into recipient animals to establish pregnancies. Difficulty in maintaining primary somatic cells in culture for a sufficient period to allow pre‐screening and expansion prior to performing SCNT constitutes an additional drawback. Moreover, offspring derived from SCNT often have developmental defects, which preclude analysis of the intended phenotype in the first generation. Finally, SCNT is technically challenging and resource‐intensive, and therefore remains unavailable to all but specialized laboratories. Even in established labs, the outcome of SCNT is unpredictable. However, efficiencies of SCNT are steadily rising owing to improvements in culture regimes 64 and SCNT remains a major driver for generating gene‐edited and other genetically engineered livestock, especially pigs.

An alternative to SCNT is performing gene editing directly in embryos. The cocktail of editors and targeting vectors (for HDR) can be microinjected into the cytoplasm or pronucleus of zygotes (Figures 2A and 2B). In pigs, the pronucleus is not readily visible, and therefore cytoplasmic injections remain the preferred route for generating edited animals. The procedure is surprisingly simple. Embryos at early stages can be recovered by surgical flush from the oviduct of donor embryos. The mRNA for editors can be injected into the cytoplasm of one‐cell zygotes (Figure 2B), which are then transferred into the oviducts of synchronized recipients to generate edited pigs 65, sheep, and cattle 42.

There are a few limitations in performing embryo injections: embryonic losses due to the toxicity of editors; incidence of mosaicism of edits; unpredictability of the percentage of edited animals; and the number of different genotypes produced. For example, as noted by Lillico *et al* 65, a wide distribution of genome edits is identified in the progeny of zygote injections. A significant number of offspring from embryo editing procedures by TALENs and ZFNs have been found to be wild type, with some edited animals carrying mutations that are in‐frame and few individual animals carrying more than one mutation (mosaic), thereby confounding the investigation of phenotype in the first generation 65. Refinements to these methods are being devised. In a more recent experiment, all 18 piglets generated by CRISPR injections across three pregnancies were found to be edited (Telugu *et al*, unpublished results). Given these findings, the lack of edited progeny is less of a concern; however, in‐frame mutations and mosaic genotypes remain a limitation. The field excitingly awaits success with oligo‐based HDR, which offers considerable control over the resulting genotype.

## New large animal models of disease

Gene editors now allow the precise engineering of animal disease models. If a mutation can occur in nature, it can be copied by use of the gene editors. Thus, mutations in the human genome known to be causative or associated with a human pathology can be replicated in the genome of an animal. Gene editing technology in large animals has only been made possible over the last few years and to date, only a few studies have been initiated (Table 1). We anticipate this to change dramatically over the next few years. To illustrate the potential, we have listed the range of genetic mutations possible, giving examples of disease models that could be engineered.

**Table 1:** Gene‐edited (mini‐)pigs addressing human disease

Gene(s)	Editor	Route	Reference
**NHEJ**			
*PPARγ*	ZFN	SCNT	121
*α 1,3GT*	ZFN	SCNT	107
*LDLR*	TALEN	SCNT	39
*α 1,3GT*	ZFN	SCNT	113
*CMAH*	ZFN	SCNT	110
*IL2RG*	ZFN	SCNT	118
*α 1,3GT CMAH*	ZFN	SCNT	114
*α 1,3GT*	TALEN	SCNT	120
*α 1,3GT*	ZFN	SCNT	106
*RAG1*	TALEN	SCNT	109
*RAG2*	TALEN	SCNT	109
*RAG2*	TALEN	SCNT	111
*DJ‐1*	TALEN	SCNT	122
*SLA‐1*, *2*, *3*	CRISPR/Cas9	SCNT	115
*CD1d*	CRISPR/Cas9	SCNT	119
*CD1d*	CRISPR/Cas9	CPI	119
*TYR*	CRISPR/Cas9	SCNT	123
*PARK2*, *PINK1*	CRISPR/Cas9	SCNT	123
*IgM*	CRISPR/Cas9	SCNT	124
*PKD1*	ZFN	SCNT	108
*α 1,3GT*, *CMAH*, *iGb3S*	CRISPR/Cas9	SCNT	112
*Npc1l1*	CRISPR/Cas9	CPI	117
**HDR**			
*CMAH*	ZFN	SCNT	110
*APC*	TALEN	SCNT	116

### Frame shift mutation

Conceptually, the simplest mutation would result from a NHEJ event at a target genetic locus. In most cases, the target would be within the coding region of a gene and this would result in a frame‐shift event with regard to the gene's open reading frame. The likely outcome would be to bring a premature stop codon into frame and produce a truncated protein. This is exactly what has been achieved for the porcine RELA gene in a project addressing resilience to viral disease 65. In this case, the final exon of the gene was targeted, with the prediction being that the truncated RELA protein would retain some function; alternatively, targeting NHEJ to an earlier exon often leads to nonsense‐mediated decay of the transcript and hence functional knockout of the protein 66.

An example disease where this strategy could be applied is Crohn's disease. A frameshift mutation (3020insC) in NOD2 thought to reduce the NFκB‐induced innate immune response in these patients is associated with susceptibility to Crohn's disease 67. Through gene editors, this frameshift could be easily produced in an animal model. Given the association of this frameshift mutation in a number of diseases, from cancer 68 to sepsis 69, and associated with treatment regime prognosis 70, such an engineered animal model could have wide‐ranging utility.

### Allele swap

A more elegant mutation strategy involving HDR would be to engineer an allele swap. In this scenario, a disease associate allelic variant would be produced. This has already been achieved for cystic fibrosis (CF) using ‘old’ transgenic methodology, with the resulting animals demonstrating research and translational opportunities. The most common CF‐associated mutation is CFTR ΔF508; however, when this mutation was engineered into mice 71, they failed to replicate the CF pathology; when the same mutation was engineered into pigs, a range of CF pathology was observed 72, 73, 74. It is now timely to produce large animal models carrying other CF‐associated alleles. Although engineered CF pigs are available, some believe that sheep may represent an alternative model species 75, and sheep are currently central to the development of gene therapy strategies to mitigate this disease 76, 77. Although some differences in lung anatomy and biochemistry manifest for these species in comparison to humans 78, 79, the inability of rodent models to replicate the disease justifies the research effort.

Engineering models provide information on the disease and offer a translational model to develop new and effective therapies. This includes gene therapy, with CF seen as potentially an early success story. Gene editors have been used to successfully correct and restore function to the CFTR in human cells *in vitro* 80. In animals, we have the opportunity to engineer disease pathology and then validate the treatment prior to human trials.

### Repeat sequence expansion

A more challenging allele swap is represented in diseases caused by expansion of unstable trinucleotide repeats 81. Spinocerebellar ataxia type 1 is an autosomal dominant neurodegenerative disorder. The neuropathology involves selective neuron loss from the cerebellum and disease severity reflects expansion size of the highly polymorphic CAG repeat 82. A similar situation exists for Huntington's disease, where expansion of a polyglutamine tract encoded within exon 1 of the huntingtin gene (*HTT*) results in pathology. The opportunity for large animal models of this disease has been championed 11, with initial progress achieved through microinjection of sheep zygotes with an expression cassette encoding the huntingtin gene with an expanded CAG repeat 83. Excitingly, it is possible that such animal models of neurodegenerative disease can be used to develop effective treatment for different protein misfolding diseases 84.

### Exon/domain deletion

The differences between the different gene editors direct their application. For instance, the use of CRISPR/Cas9 with two guide RNAs allows the efficient deletion of DNA sequence between the two guides, even over relatively large genomic distances 85. Proteins often consist of several peptide domains, each of which is often encoded by a single exon within the gene, and sequence deletion can be associated with some human diseases; deletion of exon 9 of the presenilin 1 gene (*PSEN1*) causes some forms of Alzheimer's disease 86. Although a number of mouse models of Alzheimer's disease exist 86, 87, 88, 89, 90, 91, 92, treatments developed in these rodent models have had very limited impact in the clinic 93, presumably linked to the large differences in brain architecture between humans and these rodents. Large animal models should be able to contribute to the pressing need for better translation strategies.

### Chromosomal translocation

Chromosomal translocations are severe genome rearrangements and those that are not lethal are often associated with pathology. Examples include Burkitt's lymphoma 94 and acute myeloid leukaemia 95, for which successful treatment is an ongoing challenge 96. Although conceptually similar in strategy, chromosomal translocations present a bigger challenge for gene editor technology than sequence deletion. Translocation has been achieved in cells 97 *in vitro* but remains to be demonstrated in animals.

### Monkeys and primates

Although all human disease deserves research attention, it is for neural disorders that we predominantly justify the use of non‐human primates 13. Gene editors can and have been used in monkeys 98, for example CRISPR/Cas9 disruption of the dystrophin gene (*DMD*) in the rhesus macaque 99. We can anticipate more research activity to rapidly emerge.

In the illustrative examples above, the desire is to engineer into the genome a disease mutation. This will require the design and production of gene editors, usually in conjunction with a DNA template carrying the desired mutation, targeting a precise, predetermined genetic locus. This approach and resources would provide valuable information for the opposite goal, that of correcting a deleterious mutation. Such corrective strategies, whilst unlikely to be applied to livestock in agriculture, could have utility for some domesticated pet species and play to the international discussion on whether gene editors should be used on the human germline 100, 101, 102, 103.

## The next 5 years

The continuously increasing need for new disease treatments must be juxtaposed against the current disappointing rate of new drugs developing through to clinical use. There are many reasons for the high attrition rate during drug discovery 104, with the paucity of reliable animal models being only one. However, utility and ease of use indicate that gene editing technology will have a role in meeting this need, overcoming the historically technical and laborious challenges of transgenesis 105, thus providing a strategy to broaden the repertoire of useful animal disease models significantly beyond that currently available. Because of the ability to make many disease models, this technology should go on to affect more than the highest profile diseases that often attract the attention of funding agencies.

We now face a research environment where the most appropriate animal species can be utilized to bridge the ‘bench to bedside’ development gap. For some diseases this may be laboratory animals but for others it will be livestock. The choice of species will depend on both comparative biology and economic factors. In addition to the use of mini‐pigs continuing, we anticipate expanding interest in standard pigs as well as sheep, and the expansion of studies using genome‐engineered primates. Indeed, there is no technical reason why gene‐editing tools could not be applied to any species for which sufficient embryology expertise exists.

The ability to engineer the same disease mutation into several species and then compare the different models against the observed human pathology is a powerful strategy to advance new treatments. It will, however, require the appropriate handling and analysis facilities for a range of differently sized animal species plus the scientific skill base to perform these comparative studies – and central to this will be pathology.

## Author contribution statement

All co‐authors wrote the manuscript, generated the figures, and had final approval of the submitted and published versions.
